# Endothelial glycocalyx degradation and its association with clinical outcomes and host response aberrations in community-acquired pneumonia across different care settings

**DOI:** 10.1186/s13054-025-05719-7

**Published:** 2026-01-27

**Authors:** Hui Wang, Erik H. A. Michels, Mingyang Cai, Joe M. Butler, Justin de Brabander, Tom D. Y. Reijnders, Sebastiaan C. Joosten, Timothy E. Sweeney, Alex R. Schuurman, Tjitske S. R. van Engelen, Bastiaan W. Haak, Xanthe Brands, Renée A. Douma, Olaf C. Cremer, Hessel Peters-Sengers, W. Joost Wiersinga, Tom van der Poll

**Affiliations:** 1 Center for Infection and Molecular Medicine (CIMM), Amsterdam UMC, University of Amsterdam, Amsterdam, The Netherlands; 2 Data Science and Epidemiology, Amsterdam UMC, University of Amsterdam, Amsterdam, The Netherlands; 3 Inflammatix Inc, Sunnyvale, CA USA; 4 Department of Internal Medicine, Flevo Hospital, Almere, The Netherlands; 5 Department of Intensive Care, UMC Utrecht, Utrecht, The Netherlands; 6 Division of Infectious Diseases, Amsterdam UMC, University of Amsterdam, Amsterdam, The Netherlands

**Keywords:** Syndecan-1, Biomarkers, Endothelium, Community-acquired pneumonia, Transcriptome

## Abstract

**Background:**

Endothelial glycocalyx degradation has been implicated in the pathogenesis of sepsis. Previous studies linked elevated plasma syndecan-1, an established biomarker of glycocalyx disruption, to mortality in sepsis. We aimed to determine the association of plasma syndecan-1 levels with clinical outcomes and host response changes in patients with community-acquired pneumonia (CAP) with various disease severities.

**Methods:**

We included CAP patients upon presentation in three care settings: emergency department (ED), general ward, and intensive care unit (ICU). We stratified patients in a Normal-Syndecan-1 and an Elevated-Syndecan-1 group based on syndecan-1 levels measured in outpatient controls without infection, and measured 32 biomarkers reflective of five pathophysiological domains involved in sepsis immunopathology: coagulation activation, endothelial cell activation and dysfunction, cytokines, neutrophil degranulation, systemic inflammation and organ damage. We analyzed blood transcriptomes to obtain insight in changes in host response pathways in circulating leukocytes related with glycocalyx disruption.

**Results:**

We included 50 non-infectious control patients and 384 CAP patients, with samples collected from 95 in the ED, 124 after admission to the general ward, and 165 after admission to the ICU. The Elevated-Syndecan-1 group showed significantly reduced 30-day survival compared to the Normal-Syndecan-1 group (log-rank p < 0.05). The relationship between syndecan-1 (continuous variable) and 30-day mortality was non-linear and independent of comorbidities and disease severity. Most biomarkers were already strongly elevated in the Normal-Syndecan-1 group relative to non-infectious controls, spanning all pathophysiological domains. All biomarkers showed further increases in the Elevated-Syndecan-1 group relative to non-infectious controls, which was also reflected in direct comparisons between the Normal-Syndecan-1 and Elevated-Syndecan-1 groups. Gene set enrichment analysis of blood leukocytes indicated a link between elevated syndecan-1 and increased expression of genes involved in extracellular matrix organization and hemostasis.

**Conclusions:**

Glycocalyx degradation, as measured by plasma syndecan-1, shows a non-linear association with mortality in patients with CAP. Key systemic host response changes implicated in sepsis pathogenesis occur prior to detectable glycocalyx degradation in this population.

**Supplementary Information:**

The online version contains supplementary material available at 10.1186/s13054-025-05719-7.

## Introduction

Community-acquired pneumonia (CAP) has a profound global impact and remains the leading cause of sepsis [1, 2]. According to the latest 2021 Global Burden of Diseases study, lower respiratory tract infections account for over 2.18 million deaths worldwide [1]. The Sepsis-3 definition implies that dysregulation of the host immune response is the primary factor that transforms uncomplicated CAP into sepsis [3]. This deleterious response involves a complex interplay between inflammation, coagulation, and endothelial cell activation [2, 4].

The endothelial glycocalyx is a gel-like layer lining the vascular endothelium, consisting of proteoglycans (such as syndecan-1), glycosaminoglycans (such as hyaluronan), glycoproteins, and glycolipids [5–8]. In a healthy state, the glycocalyx plays a crucial role in regulating vascular permeability and leukocyte adhesion, and exhibits anticoagulant and anti-adhesion properties [5, 9]. In sepsis, however, the glycocalyx undergoes significant degradation, leading to edema, heightened leukocyte adhesion, platelet aggregation and intensified tissue injury [6, 7, 9–11]. During disruption of the glycocalyx components of this surface layer are released into the bloodstream of which plasma syndecan-1 is the most established marker to assess the extent of glycocalyx degradation [5, 12–14].

Increased plasma syndecan-1 concentrations are associated with increased fluid requirements, need for renal replacement therapy, disseminated intravascular coagulation, and higher mortality in patients with sepsis [5, 13, 15–17]. Noteworthy, syndecan-1 has been shown to outperform other glycocalyx-related markers, such as endocan and hyaluronan, in predicting these adverse outcomes [13, 18–20]. Preservation of the glycocalyx in animal models demonstrated potential for therapeutic interventions in sepsis [21–29]. This prior research highlights the prognostic value of glycocalyx degradation in sepsis and its potential as a viable target for therapy.

Previous research primarily focused on the association between glycocalyx disruption and clinical outcomes in sepsis [5, 17], while its association with host response aberrations mostly involved small patient cohorts and a minimal number of biomarkers (typically fewer than five) [15, 18, 20, 30–32]. This narrow scope leaves a significant gap in understanding the timing and extent of glycocalyx disruption in CAP patients with varying disease severities, and the implications of glycocalyx degradation for other pathophysiological changes relevant for the pathogenesis of severe infection. This study addresses these gaps by examining a large cohort of CAP patients across disease severities and care settings (emergency department (ED), general ward and intensive care unit (ICU)). We measured 32 plasma biomarkers reflective of coagulation activation, endothelial cell (dys)function, cytokines, neutrophil degranulation, systemic inflammation and organ damage. By stratifying patients based on syndecan-1 levels and incorporating blood transcriptomic data, we aimed to provide a comprehensive understanding of host response changes associated with glycocalyx disruption.

## Methods

### Study design and population

We analyzed the data of three independent prospective observational studies in the Netherlands, conducted in different care settings: the OPTIMACT study (ED, Dutch Trail Register identifier NTR6163,. Registered December 6, 2016) [33], the ELDER-BIOME study (general ward, Clinicaltrials.gov identifier NCT02928367, Registered October 1, 2016) [34, 35], and the MARS project (ICU, ClinicalTrials.gov identifier NCT01905033, Registered January, 2011) [36]. Ethical approval for each study was granted by the local ethics committees. In the OPTIMACT and ELDER-BIOME studies trained clinicians screened potential participants from October 2016 to July 2018 in one academic (Amsterdam UMC, Amsterdam, the Netherlands) and three regional hospitals (BovenIJ Hospital, Amsterdam; Flevo Hospital, Almere; Spaarne Gasthuis, Haarlem; all in the Netherlands); written informed consent was obtained from all patients. The MARS project included all ICU admissions at two teaching hospitals (Amsterdam UMC and UMC Utrecht) between January 2011 and January 2014, using an opt-out method approved by the ethics committee. Patients who met each of the following CAP criteria were included: (1) respiratory symptoms such as a new cough or sputum production, chest pain, dyspnea, tachypnea, abnormal lung examination, (2) systemic manifestations like documented fever or hypothermia, leukocytosis or leukopenia, and (3) evident chest X-ray or CT scan findings of new infiltrates, consolidation, or pleural effusion. Cases suspected of aspiration or hospital-acquired pneumonia were excluded. The Modified Early Warning Score (MEWS) was used to assess disease severity; MEWS is a simple bedside tool that uses vital signs (heart rate, systolic blood pressure, respiratory rate, oxygen saturation, temperature, level of consciousness) to identify hospitalized patients at risk of clinical deterioration [37]. Definitions of comorbidities can be found in the Additional file 1. Additionally, as part of the ELDER-BIOME study, we included non-infectious outpatient clinic controls, with similar comorbidities, age and sex distribution; for this all patients were eligible if their medical doctor ordered a routine blood draw, and were free of acute disease including infection. All controls provided written informed consent.

### Biomarker assays

We collected EDTA-plasma samples from patients either immediately upon arrival at the ED or within the first 16 h of general ward or ICU admission. We analyzed 32 biomarkers (Additional file 1, Table S1) using the Luminex 200 (R&D Systems, Minneapolis, MN) and BioPlex 200 system (BioRad, Hercules, CA). To ensure consistent measurements, we determined all biomarker levels in a single run using the same batch of reagents, with all samples randomized across assay plates. All samples were kept frozen at −80 °C until analyzed. Biomarkers were stratified according to pathophysiological domain based on literature [38–43].

### Whole-blood transcriptomic analyses

Blood for RNA analyses was collected in Paxgene tubes (Becton Dickinson). Whole blood transcriptomes from the MARS cohort were analyzed using Affymetrix U219 arrays as described in detail [36]. For the ELDER-BIOME and OPTIMACT projects, RNA was processed with the QIAseq Stranded Total RNA Library Kit, including rRNA and globin depletion. Libraries were sequenced on a NovaSeq 6000, and data was aligned to the human genome (GRCh38) using STAR v2.7.9. Only samples with RNA integrity number ≥ 5 were included. Batch and platform correction across datasets was achieved using the Coconut R package [44].

### Statistical analysis

All analyses were performed in R (version 4.4.1) (R Core Team 2024, Vienna, Austria). Biomarker data was log-transformed. Patients were stratified into two groups based on plasma syndecan-1 levels, regardless of care setting: the Normal-Syndecan-1 group and the Elevated-Syndecan-1 group. The Normal-Syndecan-1 group was selected to have similar levels to a group of non-infected controls. Specifically, we identified a threshold cut-off of syndecan-1 such that the mean level in the Normal-Syndecan-1 subgroup was equivalent to the mean level observed in the non-infected control group. To determine this threshold, we employed an iterative, trial-and-error approach which found a cutoff of 3037.9 pg/mL, which translates to a z-score of 1.43 in the non-infectious control sample (i.e. this syndecan-1 level is 1.43 standard deviations higher than the mean in this sample). We also performed a secondary sensitivity analysis using a z-score of 1.96 which translates to the upper 95% confidence interval.

We assessed data distributions using histograms and quantile–quantile plots. Between-group comparisons of non-normally distributed continuous variables were performed using the Wilcoxon rank-sum test. Differences in 30-day survival rates between syndecan-1 groups were visualized using Kaplan–Meier curves. In mortality adjusted models, we accounted for potential confounders of glycocalyx disruption with the aim to isolate effect of glycocalyx degradation. The adjusted model included demographics (age, sex), vascular comorbidities (prior myocardial infarction, cerebrovascular disease, chronic kidney disease, diabetes), and immunosuppression. Given the potential causal rather than confounding relationships between disease severity at admission and glycocalyx degradation, we performed an independent adjusted model with MEWS as an additional covariate. To account for baseline risk differences across sampling locations, we included care setting at sampling as an additional covariate. When examining associations of syndecan-1 on a continuous scale with 30-day mortality, we first accessed linearity of this relationship with a restricted cubic spline with 3 inner knots at default locations followed by an analysis of variance to test for the significance of the non-linear terms. We also conducted an exploratory cut-point analysis using maximally selected rank statistics (surv_cutpoint in R) to identify the syndecan-1 level most strongly associated with 30-day survival. To quantify differences on a single biomarker level between syndecan-1 groups, we used Hedges’ g [45], a commonly used effect size measure. Linear regression models were used with log-transformed biomarker concentrations as the outcome and patient group as the main predictor. Analyses were performed separately with adjustment for plasma creatinine (log-transformed) and for immunosuppression. In a fully adjusted specification, we additionally included demographics (age, sex), vascular comorbidities (prior myocardial infarction, cerebrovascular disease, chronic kidney disease, diabetes), MEWS and care setting in the covariate set. For the transcriptomic analysis, we used the limma package in R with empirical bayes correction [46]. For functional enrichment, we specifically focused on Reactome pathways directly related to syndecan-1: "Hemostasis" (R-HSA-109582) and "Extracellular Matrix Organization" (R-HSA-1474244) [47].

All p-values were adjusted for multiple testing using the Benjamini–Hochberg method [48]. All clinical characteristics and biomarkers demonstrated less than 5% missingness and were classified as missing at random. The limited number of missing platelet counts (2.60% missing), heart rate (0.78% missing), respiratory rate (0.78%), and core temperature (1.95%) were imputed using the MICE package using the CART method. For details on each of the statistical analyses, see Additional file 1.

## Results

### Baseline characteristics of patients and mortality

We included 50 non-infectious control patients (Additional file 1, Table S2) and 384 patients with community-acquired pneumonia (CAP). Of CAP patients, 95 (24.7%) were sampled in the ED, 124 (32.3%) were sampled within 16 h after admission to a general ward, and 165 (43.0%) were sampled within 16 h after admission to the ICU. Of the 95 ED patients, 37 (38.9%) did not need hospital admission. Patients were stratified into two groups based on a syndecan-1 cutoff of 3037.9 pg/mL (see Methods): the Normal-Syndecan-1 group (n = 158, 41.1%) and the Elevated-Syndecan-1 group (n = 226, 58.9%; Table 1). These two groups were generated to allow for comparison of host response changes in the presence of a relatively intact glycocalyx versus a damaged glycocalyx. Syndecan-1 levels did not differ between the Normal-Syndecan-1 group (median 2220.3 pg/mL, interquartile range [IQR] 1753.5–2645.4) and outpatient controls without infection (2143.6 pg/mL, IQR 1888.0–2536.2 pg/mL); as expected the Elevated-Syndecan-1 group had significantly higher syndecan-1 concentrations (median 4797.3 pg/mL, IQR 3820.2–6497.8; p < 0.001 for comparison with both other groups; Additional file 1, Figure S1). ICU patients made up 53.5% of the Elevated-Syndecan-1 group and 27.8% of the Normal-Syndecan-1 group. In the Elevated-Syndecan-1 group, 46.5% of patients came from the ED or the ward. This pattern suggests that elevated syndecan-1 levels are present across all care levels, not just in critically ill patients. The two groups were similar in age, sex and body mass index. The Elevated-Syndecan-1 group had higher rates of immunosuppression (28.3% vs. 13.9%, p = 0.001), and chronic kidney disease (18.1% vs. 1.3%, p < 0.001). Other comorbidities were equally distributed. Patients in the Elevated-Syndecan-1 group had higher plasma creatinine (median 110 vs 81 μmol/L, p < 0.001) and urea levels (median 9.50 vs 5.95 mmol/L, p < 0.001). Platelet counts, white blood cell counts, and bilirubin levels were similar. Disease severity was greater in the Elevated-Syndecan-1 group, reflected by higher MEWS (median 6.00 vs 4.00, p < 0.001).

**Table 1:** Baseline characteristics and outcome of patients with community-acquired pneumonia stratified according to plasma syndecan-1 concentrations upon presentation

n	Normal-Syndecan-1 group	Elevated-Syndecan-1 group	p-value
	158	226	
Syndecan-1 (pg/ml), median [IQR]	2220.3 [1753.5, 2645.4]	4797.3 [3820.2, 6497.8]	< 0.001
Sample location			< 0.001
Emergency department, n (%)	67 (42.4)	28 (12.4)	
General ward, n (%)	47 (29.7)	77 (34.1)	
Intensive care unit, n (%)	44 (27.8)	121 (53.5)	
Demographics			
Age, years, median [IQR]	66.0 [51.3, 74.8]	65.0 [52.0, 75.0]	0.98
Sex, male, n (%)	89 (56.3)	133 (58.8)	0.699
Body mass index, median [IQR]	24.9 [22.3, 27.7]	24.1 [21.4, 27.6]	0.214
Comorbidities			
COPD, n (%)	52 (32.9)	44 (19.5)	0.004
Congestive heart failure, n (%)	11 (7.0)	19 (8.4)	0.744
Prior myocardial infarction, n (%)	22 (13.9)	22 (9.7)	0.269
Cerebrovascular disease, n (%)	16 (10.1)	19 (8.4)	0.692
(Prior) malignancy, n (%)	39 (24.7)	58 (25.7)	0.922
Immunosuppression, n (%)	22 (13.9)	64 (28.3)	0.001
Chronic kidney disease, n (%)	2 (1.3)	41 (18.1)	< 0.001
Diabetes, n (%)	30 (19.0)	58 (25.7)	0.159
Severity on admission			
MEWS, median [IQR]	4 [2, 7]	6 [3, 11]	< 0.001
Routine laboratory markers median [IQR]			
Platelet counts (× 109/L)	224 [170, 281]	200.0 [132, 300]	0.237
Leukocyte counts (× 109/L)	12.05 [9.20, 15.15]	11.40 [6.40, 16.40]	0.233
Creatinine (µmol/L)	81 [57, 102]	110 [78, 176]	< 0.001
Urea (mg/dL)	5.95 [4.47, 8.60]	9.50 [6.45, 15.80]	< 0.001
Bilirubin (µmol/L)	9 [6, 17]	10 [6, 16]	0.474
Outcome			
30-day mortality, n (%)	13 (8.2)	40 (17.7)	0.013

IQR: Interquartile range; COPD: Chronic obstructive pulmonary disease; MEWS: Modified Early Warning Score.

**Fig. 1:**
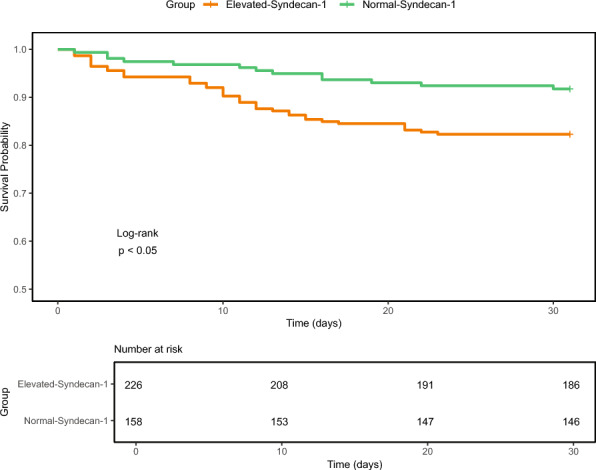
30-day Kaplan–Meier survival curves

The Elevated-Syndecan-1 group had a higher 30-day mortality (17.7% vs 8.2% in the Normal-Syndecan-1 group, p = 0.013, Table 1; Kaplan–Meier analysis shown in Fig. 1, log-rank p < 0.05). Syndecan-1 was also associated with 30-day mortality when modelled as a continuous variable (p < 0.0001). The relationship between syndecan-1 and 30-day mortality was non-linear (p = 0.008; Additional file 1, Figure S2). After adjustment for demographics and comorbidities, the association remained significant (p = 0.0021). This finding held after further adjustment for MEWS (p = 0.0005) and MEWS plus care setting (p = 0.0005). Cut-point analysis yielded an optimal threshold of 4474 pg/mL, above which patients showed a substantially higher mortality risk (Additional file 1, Figure S3). In agreement with an increased risk for adverse outcomes in patients with increased syndecan-1 levels, among patients enrolled in the ED, ICU transfer after admission to the hospital occurred in 14.3% of the Elevated-Syndecan-1 group versus 4.5% of the Normal-Syndecan-1 group; among patients recruited in the ward, 10.4% of the Elevated-Syndecan-1 group vs 4.3% of the Normal-Syndecan-1 group were transferred to the ICU after their initial admission. Among patients enrolled in the ICU the requirement for vasopressors and renal replacement therapy in the first 30 days after admission was higher in the Elevated-Syndecan-1 group, while the need for mechanical ventilation did not differ between groups (Additional file 1, Table S3).

In summary, elevated syndecan-1 levels are common yet not universal in CAP patients and display a non-linear positive association with 30-day mortality, independent of comorbidities and disease severity at admission.

### Association of syndecan-1 with host response changes in other pathophysiological pathways

**Fig. 2:**
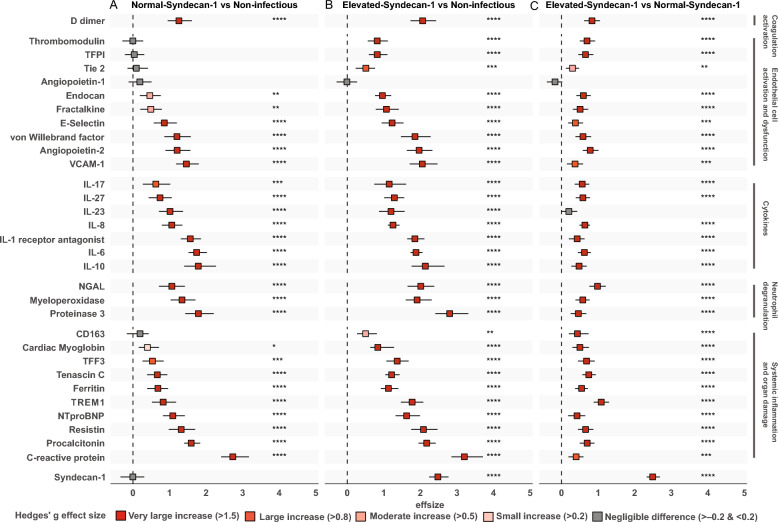
Association of syndecan-1 with individual host response biomarkers. The figure displays the differences in syndecan-1 levels, measured using Hedges’ g. Comparisons are shown for: (**A**) Normal-Syndecan-1 vs Non-infectious controls, (**B**) Elevated-Syndecan-1 vs Non-infectious controls, (**C**) Elevated-Syndecan-1 vs Normal-Syndecan-1 patients. Each square represents the Hedges’ g effect size with 95% confidence intervals. A positive value indicates higher biomarker levels in the first group of the comparison. Hedges g’ effect sizes are considered very large if > 1.5, large if > 0.8, moderate if > 0.5 and small if > 0.2 [45]. The markers are grouped by biological domains. Colors indicate the statistical significance and direction of change, based on Welch’s t-test with Benjamini–Hochberg correction: red denotes significant increases, grey indicates non-significant differences. Abbreviations: NGAL: neutrophil gelatinase-associated lipocalin; NTproBNP: aminoterminal pro-B-type natriuretic peptide; TREM1: triggering receptor expressed on myeloid cells 1; VCAM-1: vascular cellular adhesion molecule-1; TFPI: tissue factor pathway inhibitor; TFF3: trefoil factor 3

To evaluate the relationship between glycocalyx degradation and the host response, we measured 32 plasma biomarkers across five key pathophysiological domains: coagulation activation, endothelial cell activation and dysfunction, cytokines, neutrophil degranulation, systemic inflammation and organ damage [2, 38–43]. Table S4 in Additional file 1 shows plasma biomarker concentrations stratified according to group and domain. We used Hedges’ g to assess effect sizes between groups [45]. In a first analysis we compared the Normal-Syndecan-1 group with the non-infectious control group, seeking to determine which host response biomarkers were altered in CAP patients who did not have detectable glycocalyx degradation yet, as indicated by syndecan-1 levels similar to those in controls (Fig. 2A). Remarkably, the vast majority of biomarkers was already strongly elevated in the Normal-Syndecan-1 group, spanning all pathophysiological domains (Hedges’g effect sizes of > 0.8 or > 1.5 are considered large or very large respectively) [45]. This suggests that coagulation activation (D-dimer) and systemic inflammatory responses, including cytokine release and neutrophil degranulation, occur before detectable glycocalyx degradation. In addition, the strongly elevated plasma levels of soluble E-selectin, vascular cell adhesion molecule-1 and von Willebrand factor in the Normal-Syndecan-1 group suggest that endothelial cell activation is an early event relative to glycocalyx disruption in patients with CAP, while the markedly elevated angiopoietin-2 concentrations in the Normal-Syndecan-1 group suggest that endothelial barrier integrity likewise is disturbed prior to detectable glycocalyx degradation [42, 43]. Endothelial cell markers that were still in normal rage in the Normal-Syndecan-1 group were thrombomodulin, tissue factor pathway inhibitor, Tie2 and angiopoietin-1; these markers were elevated in the Elevated-Syndecan-1 group with the exception of angiopoietin-1 which remained within normal range. All other biomarkers showed further increases in the Elevated-Syndecan-1 group relative to non-infectious controls across all pathophysiological domains (Fig. 2B), as also reflected in direct comparisons between the Normal-Syndecan-1 and Elevated-Syndecan-1 groups (Fig. 2C). These results remained essentially unchanged after adjustment for plasma creatinine or immunosuppression (Additional file 1, Table S5).

In the analyses above a syndecan-1 cutoff of 3037.9 pg/mL was used to stratify CAP patients in a Normal-Syndecan-1 and Elevated-Syndecan-1 group. We performed a sensitivity analysis using a more stringent cutoff (3351.6 pg/mL; z = 1.96), which yielded results that were highly similar to our primary analysis (Additional file 1, Figure S4).

### Transcriptomic analysis of blood leukocytes reveals hemostasis and extracellular matrix pathway alterations associated with elevated syndecan-1 levels

**Fig. 3:**
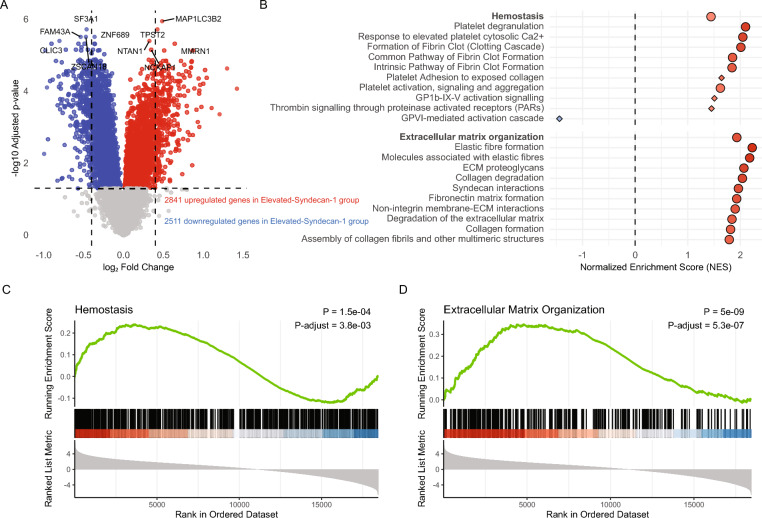
Differentially expressed genes and pathway enrichment results comparing the Elevated-Syndecan-1 and the Normal-Syndecan-1 group. (**A**) The volcano plot illustrates differential gene expression between the Elevated-Syndecan-1 and the Normal-Syndecan-1. Red points represent genes that are significantly upregulated in the Elevated-Syndecan-1 group (a total of 2841 upregulated genes), while blue points indicate downregulated genes (a total of 2511 downregulated genes). The ten most significant upregulated and downregulated genes are labelled. (**B)** The plot displays the Normalized Enrichment Score (NES) for Reactome pathways related to extracellular matrix organization and hemostasis. These pathways are organized into child pathways (depicted in lighter colors) and parent pathways (shown in darker colors). Red indicates pathways enriched in the Elevated-Syndecan-1 group; blue indicates enrichment in the Normal-Syndecan-1 group. Circles represent significant pathways (false discovery rate < 0.05), diamonds represent non-significant ones. Larger dots reflect stronger statistical support. (**C**) Enrichment plot for the Reactome "Hemostasis" pathway. The running enrichment score (represented by the green line) indicates the extent of expression of pathway-related genes at the top of the ranked gene list, with the peak score signifying the most significant enrichment. Vertical black lines denote the rank positions of individual genes within the pathway. The heatmap below the enrichment plot illustrates the ranked list metric, where red shading indicates higher expression and blue shading indicates lower expression in the Elevated-syndecan-1 group. (**D**) Enrichment plot for the Reactome "Extracellular Matrix Organization" pathway

The glycocalyx can impact the function of circulating leukocytes, particularly in the context of adhesion and inflammation [6–8, 43]. Thus, we sought to determine the association between glycocalyx disruption and changes in the transcriptome of blood leukocytes. First, we analyzed differentially expressed genes between the Elevated-Syndecan-1 and Normal-Syndecan-1 groups in an untargeted approach using all genes. We identified 2841 upregulated and 2511 downregulated genes when comparing the Elevated-Syndecan-1 to the Normal-Syndecan-1 group (Fig. 3A). Notably, several of the most significantly altered genes were associated with hemostasis and extracellular matrix interactions; i.e., notable upregulated genes in the Elevated-Syndecan-1 group were *MMRN1* (encoding multimerin 1, involved in platelet function), *TPST2* (tyrosylprotein sulfotransferase 2), and *MAP1LC3B2* (microtubule-associated protein linked to autophagy and cellular stress responses). In contrast, *FAM43A* (family with sequence similarity 43 member A), *SF3A1* (splicing factor 3a subunit 1), and *ZSCAN18* (zinc finger and SCAN domain containing 18) were among the most significantly downregulated genes in this group.

Considering these findings and the crucial role of the glycocalyx in maintaining vascular integrity and regulating leukocyte adhesion and hemostasis [5–7], we specifically targeted these pathways (“hemostasis” and “extracellular matrix organization”), in a gene set enrichment analysis using the Reactome database [47] (Fig. 3B). Nearly all sub-pathways within the “extracellular matrix organization” category were significantly upregulated, including elastic fiber formation, extracellular matrix proteoglycans, degradation of the extracellular matrix, collagen formation, non-integrin membrane–extracellular matrix interactions, and fibronectin matrix formation. Similarly, upregulation of pathways related to platelet activation and clot formation—such as the clotting cascade, platelet activation, and signaling—was observed within the “hemostasis” domain. Collectively, these findings support the hypothesis that glycocalyx degradation in CAP, as reflected by elevated plasma syndecan-1, may be associated with gene programs indicative of extracellular matrix remodeling and a pro-thrombotic state.

We performed a sensitivity analysis in which the Normal-Syndecan-1 and Elevated-Syndecan-1 groups were determined using a more stringent cutoff of 1.96 standard deviations above the non-infectious group mean, which yielded similar results (Additional file, Figure S5). A further exploration of the GSEA results highlighted that, while most genes in “extracellular matrix organization” and “hemostasis” pathways were upregulated in the Elevated-Syndecan-1 group, some genes in the "hemostasis" pathway were downregulated (Fig. 3C). An overrepresentation analysis revealed that these down-regulated genes were particularly related to "GPVI-mediated activation cascade" and the "effects of PIP2 hydrolysis" (Additional file 1, Table S6), potentially indicating reduced platelet reactivity concurrently with the previously identified pro-coagulant environment.

To illustrate representative gene-level changes in these pathways, we visualized the top five up- and downregulated genes for each key pathway (“hemostasis” and “extracellular matrix organization”) in the Additional file, Figure S6.

## Discussion

The glycocalyx is a gel-like structure covering endothelial cells that plays a crucial role in maintaining vascular homeostasis. Earlier studies that investigated the integrity of the glycocalyx in infection were restricted to patients with sepsis, and provided limited information about associations with other host response aberrations [15, 18, 20, 30–32]. The present study reports a comprehensive analysis of glycocalyx disruption, as measured by plasma syndecan-1 levels, in CAP patients across different care settings, offering novel insights into its relationship with alterations in distinct host response pathways. Based on plasma syndecan-1 levels in non-infectious outpatient controls we divided CAP patients into those with normal versus elevated syndecan-1 concentrations. Strikingly, CAP patients in the Normal-Syndecan-1 group demonstrated marked elevations in biomarkers reflective of key pathophysiological domains involved in the pathogenesis of sepsis, suggesting that glycocalyx disruption is a relatively late phenomenon in the spectrum of host response aberrations.

Previous studies reported an association with plasma syndecan-1 levels and mortality in critically ill patients with sepsis [5, 13, 15–17]. In agreement, in the CAP cohort included in the present investigation, entailing patients with highly diverse disease severities, a strong association was apparent between elevated syndecan-1 levels and 30-day mortality, which persisted after adjusting for baseline characteristics including disease severity and care setting. The non-linear relationship between syndecan-1 and mortality suggests a potential threshold, where glycocalyx degradation beyond a certain point may lead to a sharp increase in mortality risk. A cut-point analysis identified 4474 pg/mL as the syndecan-1 level most strongly discriminating survivors and non-survivors at day 30, although this value should be considered exploratory, requiring external validation. Of note, the finding that many host response abnormalities were already evident in patients with normal syndecan-1 levels could indicate that glycocalyx degradation may not be a primary driver of mortality, but rather that plasma syndecan-1 serves as a surrogate marker for poor outcomes.

The glycocalyx is considered to play a critical role in inhibiting coagulation activation by serving as an anticoagulant surface through binding molecules like antithrombin and tissue factor pathway inhibitor [5, 6, 9]. Glycocalyx degradation is believed to amplify coagulation activation through loss of this anticoagulant function [5, 6, 9]. We here show that plasma D-dimer levels are strongly elevated in the Normal-Syndecan-1 group, suggesting that in CAP patients coagulation activation occurs in spite of a relatively intact glycocalyx. The finding that a variety of inflammatory responses, including the release of proinflammatory cytokines and neutrophil degranulation, were clearly activated in the Normal-Syndecan-1 group provides in vivo evidence for laboratory studies indicating that excessive inflammation is a driving factor in disruption of the glycocalyx [8].

The transcriptomic analysis of blood leukocytes uncovered alterations in the expression of genes related to extracellular matrix interactions and hemostasis in patients with high syndecan-1 levels, signifying responses of circulating leukocytes that are biologically compatible with glycocalyx disruption [5–8]. These results suggest that glycocalyx degradation contributes to disease progression through its effects on other host response pathways, including leukocyte functions. Nonetheless, these transcriptome data should be interpreted with caution considering the lack of mechanistic validation.

Our study has strengths and limitations. We measured 32 protein biomarkers and analyzed the transcriptome of circulating leukocytes to provide insight into key host response pathways in a large cohort of CAP patients with highly diverse disease severities, ranging from mild (presented at the ED and sent home after treatment initiation) to severe (sepsis with associated organ failure treated at the ICU). Nevertheless, our study was restricted to CAP and hospitals in the Netherlands, and the findings may not be generalizable to other infections or geographic regions. Our study was cross-sectional and observational, providing a snapshot of host responses at a single point. We specifically aimed to distinguish between host response patterns in patients with a relatively intact glycocalyx (Normal-Syndecan-1 group) and those observed only in patients showing biochemical evidence of glycocalyx injury (Elevated-Syndecan-1 group). Thereby, our goal was to explore possible connections between glycocalyx degradation and other disturbances in host immunity implicated in the pathogenesis of severe infections. These analyses should, however, be regarded as hypothesis-generating: given the study design, we cannot establish cause-and-effect relationships from our data. Additional limitations of our study are the heterogeneity of blood sampling time after presentation to the hospital and the lack of follow-up measurements. Longitudinal studies with serial measurements of syndecan-1 and other biomarkers would provide a more dynamic understanding of glycocalyx degradation in the progression of CAP. Although syndecan-1 is a widely accepted marker of glycocalyx degradation, it may not capture all aspects of glycocalyx integrity; future studies incorporating multiple glycocalyx-related markers and/or direct visualization techniques could provide a more comprehensive assessment of glycocalyx status. Other limitations of our study are the lack of information about intravenous fluids and hyperglycemia, which may influence glycocalyx shedding [49]. Plasma syndecan-1 levels may not solely reflect glycocalyx disruption, but can also be influenced by renal function [50]. Yet, adjustment for plasma creatinine did not change biomarker results stratified according to syndecan-1 group to a significant extent.

## Conclusions

Our findings demonstrate that disruption of the endothelial glycocalyx, as reflected by circulating syndecan-1 concentrations, is common in patients with CAP and associated with increased risk of death. The observation that profound host response abnormalities were already detectable in patients with preserved glycocalyx integrity suggests that glycocalyx degradation is not an initiating event but rather accompanies or follows other pathophysiological derangements. As such, our results suggest that therapies targeting glycocalyx preservation or restoration may not impact the induction of potentially harmful systemic host responses in CAP. These data underscore the importance of considering glycocalyx involvement within the broader context of host–pathogen interactions and highlight the need for future studies to clarify its temporal role in disease progression and its potential relevance as a therapeutic target.

## Supplementary Information

Additional file 1

## Data Availability

The datasets analyzed in this study can be obtained from the corresponding author upon reasonable request.

## Abbreviations

CAP: Community-acquired pneumonia
COPD: Chronic obstructive pulmonary disease
ECM: Extracellular matrix
ED: Emergency department
FAM43A: Family with sequence similarity 43 member A
GSEA: Gene set enrichment analysis
ICU: Intensive care unit
IQR: Interquartile range
MAP1LC3B2: Microtubule-associated protein linked to autophagy and cellular stress responses
MEWS: Modified early warning score
MMRN1: Multimerin 1
SF3A1: Splicing factor 3a subunit 1
TPST2: Tyrosylprotein sulfotransferase 2
ZSCAN18: Zinc finger and SCAN domain containing 18
