# Supplementary material for: Endothelial glycocalyx degradation and its association with clinical outcomes and host response aberrations in community-acquired pneumonia across different care settings
Source: Crit Care. 2026 Jan 27;30:60. doi: 10.1186/s13054-025-05719-7 (PMC12874961; doi:10.1186/s13054-025-05719-7)
Supplement: Supplementary file 1 — Additional file 1 [file 13054_2025_5719_MOESM1_ESM.docx]

**Additional file 1**

Endothelial glycocalyx degradation and its association with clinical outcomes and host response aberrations in community-acquired pneumonia across different care settings

Hui Wang*, Erik H. A. Michels*, Mingyang Cai, Joe M. Butler, Justin de Brabander, Tom D. Y. Reijnders, Sebastiaan C. Joosten, Timothy E. Sweeney, Alex R. Schuurman, Tjitske S. R. van Engelen, Bastiaan W. Haak, Xanthe Brands, Renée A. Douma, Olaf C. Cremer, Hessel Peters-Sengers, W. Joost Wiersinga, Tom van der Poll

*Equal contribution

Corresponding author: Hui Wang, hui.nerissa.wang@amsterdamumc.nl

### Table of Contents

***Supplementary tables***

Table S1 – Stratification of biomarkers in host response domains………………………………………………………6

Table S2 – Characteristics of non-infectious controls………………………………………………………………………….7

Table S3 – Organ support in the first 30 days after ICU admission in the subgroup of patients enrolled in the ICU……………………………………………………………………………………..……………………………..……………..…..…8

Table S4 – Plasma biomarker concentrations……………………………………………………………………………………..9

Table S5 – Creatinine-adjusted, immunosuppression-adjusted, and fully adjusted group differences for log-transformed biomarkers in Elevated– vs Normal–Syndecan-1 groups……………………………….….10

Table S6 – Enriched pathways from genes with negative running scores in Hemostasis……………………11

***Supplementary tables***

Figure S1 –Syndecan-1 levels across patient groups……………………..……………………..…………………………..12

Figure S2 –Relationship between syndecan-1 levels and 30-day mortality in unadjusted and adjusted models……………………..……………………..……………………………………………………………………………………………….13

Figure S3 – Kaplan–Meier survival stratified by Syndecan-1 (cut-point via maximally selected rank statistics)…………………..……………………..………………………………………………………………………………………………14

Figure S4 –Differential expression of host response biomarkers across Syndecan-1 groups………………………………………………………………………………………………………………………………………………15

Figure S5 – Differential gene expression and pathway enrichment analysis comparing the Elevated-Syndecan-1 group with the Normal-Syndecan-1 group…………………………………………………………………….16

Figure S6 – Expression levels of the 5 most upregulated and the 5 most downregulated genes in the Reactome pathways “hemostasis” and “extracellular matrix organization”.……………………………………17

## Supplementary materials

### Definition of comorbidities

Malignancy was defined as a medical history of a solid tumor, whether metastatic or not, or a hematologic malignancy. Immunosuppression was defined as a history of an organ transplant, immune deficiency or chronic use of immunosuppressants

### Transcriptomic assays

Whole blood transcriptomes in the MARS cohort were analyzed using U219 arrays (Affymetrix) as described [1]. The ELDER-BIOME and OPTIMACT project sample preparation involved isolating RNA from 3 ml samples using the PAXgene Blood miRNA Kit (Becton Dickinson) with a final elution volume of 40 µl. The QIAseq Stranded Total RNA Library Kit with QIAseq FastSelect for rRNA and globin depletion was used for library preparation. Starting with 100 ng of material, the RNA was heat fragmented and treated to reduce unwanted RNA species. cDNA synthesis followed by end-repair, adenylating, adapter ligation, PCR enrichment (18 cycles) and purification led to the final library, which was quality-checked and pooled for sequencing. Sequencing was performed on a NovaSeq 6000, using five S4 flow cells. RNA-sequencing data was processed by aligning the human genome GRCh38 with STAR v2.7.9 and GENCODE v44, applying standard parameters and gene counts for analysis. Only samples with a RNA integrity number ≥5 were deemed of sufficient quality. Data from the MARS project was integrated with that of the ELDER-BIOME and OPTIMACT projects using the Coconut R package for batch correction [2]. This established method standardizes control samples from various sources, after which the same standards are applied to patient samples. The method ensures uniformity across different datasets and provides a consistent baseline for comparing disease samples.

### Statistical and bioinformatics methods

The imputation model was set to run 20 iterations, and we used median values from the imputations to fill in the missing data for each variable. A logistic regression model was constructed using restricted cubic splines to capture the non-linear relationship between syndecan-1 levels and mortality. The model was summarized and analyzed using analysis of variance. We used the limma package in R to identify differentially expressed genes (DEGs) [3]. The resulting DEGs were filtered based on adjusted p-values using the Benjamini-Hochberg method to control for false discovery rate. We conducted the ReactomePA package to explore key biological pathways [4]. We used a ranked gene list, based on T-score, as input for the gsePathway function to identify significantly enriched Reactome pathways. From this analysis, we selected the pathways directly related to syndecan-1. The pathways "Hemostasis" (R-HSA-109582) and "Extracellular Matrix Organization" (R-HSA-1474244) were chosen as parent pathways of interest, grouping them with their relevant child pathways. For "Hemostasis", the child pathways are "Cell surface interactions at the vascular wall" (R-HSA-202733), "Platelet Adhesion to exposed collagen" (R-HSA-75892), "Platelet activation, signaling and aggregation" (R-HSA-76002), "Formation of Fibrin Clot (Clotting Cascade)" (R-HSA-140877), and " Factors involved in megakaryocyte development and platelet production" (R-HSA-983231). The child pathways for "Extracellular Matrix Organization" include "Fibronectin matrix formation" (R-HSA-1566977), "Laminin interactions" (R-HSA-3000157), and "Degradation of the extracellular matrix" (R-HSA-1474228).

### Reference

1.Scicluna BP, Klein Klouwenberg PM, van Vught LA, Wiewel MA, Ong DS, Zwinderman AH, et al. A molecular biomarker to diagnose community-acquired pneumonia on intensive care unit admission. American journal of respiratory and critical care medicine. 2015;192(7):826-35.

2.Sweeney T. COCONUT: COmbat CO-Normalization Using conTrols (COCONUT). R package version. 2017;1(2).

3.Ritchie ME, Phipson B, Wu D, Hu Y, Law CW, Shi W, et al. limma powers differential expression analyses for RNA-sequencing and microarray studies. Nucleic acids research. 2015;43(7):e47-e.

4.Yu G, He Q-Y. ReactomePA: an R/Bioconductor package for reactome pathway analysis and visualization. Molecular BioSystems. 2016;12(2):477-9.

5.Hedges LV. Distribution theory for Glass's estimator of effect size and related estimators. journal of Educational Statistics. 1981;6(2):107-28.

### Tables

**Table S1**: Stratification of biomarkers in host response domains

| Coagulation activation | Endothelial cell activation and disfunction | Cytokines | Neutrophil degranulation | Systemic inflammation and organ damage |
| --- | --- | --- | --- | --- |
| D dimer | Thrombomodulin | IL-17 | NGAL | CD163 |
|  | TFPI | IL-27 | Myeloperoxidase | Cardiac Myoglobin |
|  | Tie 2 | IL-23 | Proteinase 3 | TFF3 |
|  | Angiopoietin-1 | IL-8 |  | Tenascin C |
|  | Endocan | IL-1 receptor antagonist |  | Ferritin |
|  | Fractalkine | IL-6 |  | TREM1 |
|  | E-Selectin | IL-10 |  | NTproBNT |
|  | von Willebrand factor |  |  | Resistin |
|  | Angiopoietin-2 |  |  | Procalcitonin |
|  | VCAM-1 |  |  | C-reactive protein |
|  | Syndecan-1 |  |  |  |

Abbreviations: IL: interleukin; NGAL: neutrophil gelatinase-associated lipocalin; NTproBNP: aminoterminal pro-B-type natriuretic peptide; TREM1: triggering receptor expressed on myeloid cells 1; VCAM-1: vascular cellular adhesion molecule-1; TFPI: tissue factor pathway inhibitor; TFF3: trefoil factor 3.

**Table S2:** Characteristics of non-infectious controls

| n | 50 |
| --- | --- |
| Demographics |  |
| Age, years, median [IQR] | 70.50 [64.00, 75.00] |
| Sex, male, n (%) | 29 (58.0) |
| Body mass index, median [IQR] | 26.57 [24.76, 28.74] |
| Comorbidities |  |
| Chronic obstructive pulmonary disease, n (%) | 4 (8.0) |
| Congestive heart failure, n (%) | 2 (4.0) |
| Prior myocardial infarction, n (%) | 5 (10.0) |
| Cerebrovascular disease, n (%) | 1 (2.0) |
| (Prior) malignancy, n (%) | 10 (20.0) |
| Immunosuppression, n (%) | 4 (8.0) |
| Chronic kidney disease, n (%) | 3 (6.0) |
| Diabetes, n (%) | 6 (12.0) |

IQR: interquartile range

**Table S3**: Organ support in the first 30 days after ICU admission in the subgroup of patients enrolled in the ICU.

|  | Normal-Syndecan-1 (N=44) | Elevated-Syndecan-1 (N=121) | P |
| --- | --- | --- | --- |
| Use of Vasopressor | 22 (50.0) | 84 (69.4) | 0.034 |
| Vasopressor-free days | 29.50 [27.00, 30.00] | 28.00 [26.00, 30.00] | 0.015 |
| Use of RRT | 0 (0.0) | 22 (18.2) | 0.005 |
| RRT-free days | 30.00 [30.00, 30.00] | 30.00 [30.00, 30.00] | 0.003 |
| Use of mechanical ventilation | 36 (81.8) | 105 (86.8) | 0.583 |
| Mechanical ventilation-free days | 27.00 [23.75, 29.00] | 27.00 [21.00, 29.00] | 0.354 |
| Data are number (%) or median [interquartile ranges]. Abbreviation: RRT, renal replacement therapy. | | | |

**Table S4:** Plasma biomarker concentrations

| **Biomarker** | **Non-infectious (n = 50)** | **Normal-Syndecan-1 (n = 158)** | **Elevated-Syndecan-1 (n = 226)** |
| --- | --- | --- | --- |
| Syndecan-1, pg/ml | 2143.6 [1888.0, 2536.2] | 2220.3 [1753.5, 2645.4] | 4797.3 [3820.2, 6497.8] |
| **Coagulation activation** |  |  |  |
| D-dimer, ng/ml | 22.8 [12.4, 40.2] | 89.3 [43.0, 203.9] | 271.5 [96.3, 731.6] |
| **Endothelial cell activation and dysfunction** |  |  |  |
| Thrombomodulin, pg/ml | 6173.8 [5296.1, 7952.4] | 6304.8 [4759.9, 8491.8] | 8571.1 [6321.0, 11546.0] |
| TFPI, ng/ml | 19.3 [13.3, 23.4]] | 18.2 [11.7, 27.0] | 28.7 [20.1, 44.3] |
| Tie2, ng/ml | 21.0 [17.1, 22.8] | 21.5 [15.8, 27.9] | 25.8 [19.2, 32.3] |
| Angiopoietin-1, pg/ml | 3409.5 [1943.6, 5346.5] | 3977.7 [1890.0, 7127.5] | 3195.4 [1477.6, 6373.2] |
| Endocan, pg/ml | 643.0 [490.4, 765.5] | 753.0 [475.7, 1128.0] | 1086.5 [639.8, 2299.5] |
| Fractalkine, pg/ml | 1.4 [1.3, 2.9] | 2.9 [1.4, 5.7] | 5.7 [1.6, 9.4] |
| E-Selectin, ng/ml | 22.6 [18.2, 25.2] | 33.2 [23.5, 48.1] | 42.3 [28.6, 63.3] |
| Von Willebrand factor, pg/ml | 279.8 [182.6, 361.6] | 527.0 [350.2, 733.1] | 666.5 [472.8, 893.9] |
| Angiopoietin-2, pg/ml | 1860.7 [1203.5, 2182.1] | 3628.9 [2333.5, 5933.4] | 7136.1 [4249.8, 12599.0] |
| VCAM-1, ng/ml | 754.9 [570.4, 924.6] | 1816.3 [1070.0, 3302.6] | 2680.1 [1534.9, 4296.6] |
| **Cytokines** |  |  |  |
| IL-17, pg/ml | 4.2 [1.0, 5.4] | 7.0 [4.9, 11.6] | 12.9 [6.2, 20.0] |
| IL-27, pg/ml | 610.4 [494.5, 745.4] | 831.4 [608.5, 1054.9] | 1034.1 [831.2, 1475.5] |
| IL-23, pg/ml | 10.8 [4.3, 36.2] | 53.5 [36.2, 107.1] | 107.1 [45.1, 166.3] |
| IL-8, pg/ml | 4.1 [3.6, 5.3] | 8.7 [5.7, 13.5] | 14.2 [9.5, 31.4] |
| IL-1 receptor antagonist, pg/ml | 470.1 [341.8, 703.1] | 1858.0 [1010.8, 6632.3] | 3534.7 [1374.2, 11964.9] |
| IL-6, pg/ml | 5.6 [4.4, 7.4] | 28.2 [11.7, 97.0] | 63.1 [26.8, 392.4] |
| IL-10, pg/ml | 0.2 [0.0, 1.6] | 4.8 [2.5, 9.3] | 9.5 [4.3, 20.7] |
| **Neutrophil degranulation** |  |  |  |
| NGAL, ng/ml | 42.5 [34.7, 47.4] | 77.8 [52.5, 116.8] | 166.1 [94.4, 316.7] |
| Myeloperoxidase, ng/ml | 19.4 [14.3, 24.2] | 50.9 [32.8, 74.6] | 68.0 [45.8, 103.6] |
| Proteinase 3, ng/ml | 15.9 [11.8, 21.1] | 48.3 [31.3, 78.1] | 68.9 [47.9, 83.8] |
| **Systemic inflammation and organ damage** |  |  |  |
| CD163, ng/ml | 680.8 [427.5, 883.0] | 636.6 [419.2, 1074.9] | 1122.9 [641.4, 2058.2] |
| Cardiac Myoglobin, pg/ml | 5381.7 [4271.3, 6281.6] | 6381.6 [4154.7, 13078.3] | 12715.6 [6262.6, 29207.5] |
| TFF3, pg/ml | 1761.9 [1315.6, 2318.2] | 2202.0 [1550.4, 3079.3] | 3457.9 [2340.7, 5203.2] |
| Tenascin C, ng/ml | 11.6 [8.9, 12.8] | 13.0 [11.3, 15.1] | 16.9 [14.2, 22.5] |
| Ferritin, ng/ml | 102.8 [53.5, 185.7] | 214.6 [94.7, 414.0] | 336.7 [160.7, 755.1] |
| TREM1, pg/ml | 154.6 [125.0, 197.6] | 236.8 [172.2, 331.5] | 449.2 [292.1, 705.4] |
| NTproBNP, pg/ml | 25.0 [3.5, 67.3] | 114.3 [54.3, 310.9] | 369.1 [114.8, 1089.0] |
| Resistin, pg/ml | 6376.3 [4978.1, 8671.7] | 16234.1 [9251.0, 31358.1] | 29221.9 [16002.8, 51878.0] |
| Procalcitonin, pg/ml | 51.5 [43.2, 65.5] | 225.8 [96.2, 1195.0] | 1274.6 [203.8, 6143.2] |
| C-reactive protein, ng/ml | 922.8 [197.7, 2346.7] | 115230.0 [45142.3, 229010.0] | 187995.0 [75695.8, 338675.0] |

Data are median [interquartile range]. For abbreviations see Table S1.

**Table S5:** Creatinine-adjusted, immunosuppression-adjusted, and fully adjusted group differences for log-transformed biomarkers in Elevated– vs Normal–Syndecan-1 groups

| Biomarker | Creatinine-adjusted | | Immunosuppression-adjusted | | Full-adjusted | |
| --- | --- | --- | --- | --- | --- | --- |
|  | % change (95% CI) | BH p | % change (95% CI) | BH p | % change (95% CI) | BH p |
| **Coagulation activation** | | | | | | |
| D-dimer | 145.8% (84.0 to 228.5) | <0.0001 | 180.0% (113.8 to 266.8) | <0.0001 | 85.9% (47.6 to 134.2) | <0.0001 |
| **Endthelial cell activation and dysfunction** | | | | | | |
| Thrombomodulin | 16.6% (5.9 to 28.3) | 0.003 | 36.2% (23.3 to 50.4) | <0.0001 | 36.8% (22.7 to 52.5) | <0.0001 |
| TFPI | 49.1% (25.7 to 77.0) | <0.0001 | 51.7% (30.6 to 76.2) | <0.0001 | 37.7% (16.5 to 62.8) | 0.0003 |
| Tie2 | 28.6% (12.9 to 46.6) | 0.0004 | 16.5% (3.5 to 31.0) | 0.012 | 15.1% (1.0 to 31.0) | 0.038 |
| Angiopoietin-1 | -1.8% (-21.0 to 22.1) | **0.873** | -12.7% (-28.9 to 7.2) | **0.193** | -4.1% (-23.3 to 20.0) | **0.713** |
| Endocan | 39.3% (15.6 to 67.9) | 0.001 | 53.3% (29.4 to 81.7) | <0.0001 | 43.0% (19.2 to 71.6) | 0.0002 |
| Fractalkine | 62.2% (28.1 to 105.4) | 0.0002 | 62.1% (30.9 to 100.8) | <0.0001 | 44.0% (15.8 to 78.9) | 0.002 |
| E-Selectin | 21.7% (5.5 to 40.4) | 0.009 | 28.2% (12.7 to 45.8) | 0.0002 | 22.1% (6.2 to 40.3) | 0.006 |
| von Willebrand factor | 22.5% (9.7 to 36.8) | 0.0007 | 33.4% (20.4 to 47.9) | <0.0001 | 26.6% (13.6 to 41.1) | <0.0001 |
| Angiopoietin-2 | 59.5% (32.5 to 91.9) | <0.0001 | 85.7% (56.3 to 120.5) | <0.0001 | 57.7% (32.4 to 87.9) | <0.0001 |
| VCAM-1 | 22.1% (4.0 to 43.3) | 0.018 | 27.1% (9.6 to 47.5) | 0.002 | 30.2% (10.7 to 53.0) | 0.002 |
| **Cytokines** |  |  |  |  |  |  |
| IL-17 | 70.4% (33.8 to 117.0) | <0.0001 | 79.6% (44.3 to 123.5) | <0.0001 | 46.5% (19.5 to 79.6) | 0.0004 |
| IL-27 | 27.2% (14.2 to 41.7) | <0.0001 | 29.8% (17.5 to 43.3) | <0.0001 | 24.0% (11.4 to 38.1) | 0.0002 |
| IL-23 | -2.8% (-29.2 to 33.7) | **0.873** | 25.2% (-7.0 to 68.4) | 0.142 | 14.4% (-14.8 to 53.6) | **0.381** |
| IL-8 | 113.6% (47.2 to 210.0) | 0.0002 | 151.8% (78.6 to 255.0) | <0.0001 | 126.8% (60.8 to 219.9) | <0.0001 |
| IL-1 receptor antagonist | 36.6% (-1.2 to 88.7) | 0.067 | 82.3% (34.2 to 147.6) | 0.0002 | 41.9% (5.1 to 91.6) | 0.026 |
| IL-6 | 133.6% (49.6 to 264.8) | 0.0005 | 211.0% (105.4 to 370.9) | <0.0001 | 131.4% (53.1 to 249.8) | 0.0002 |
| IL-10 | 82.2% (26.4 to 162.6) | 0.002 | 101.6% (44.8 to 180.8) | <0.0001 | 91.1% (36.0 to 168.5) | 0.0004 |
| **Neutrophil degranulation** | | | | | | |
| NGAL | 59.0% (35.5 to 86.7) | <0.0001 | 109.8% (78.2 to 147.1) | <0.0001 | 64.3% (39.8 to 93.0) | <0.0001 |
| Myeloperoxidase | 44.2% (21.7 to 70.9) | <0.0001 | 56.9% (33.9 to 83.8) | <0.0001 | 58.8% (35.4 to 86.3) | <0.0001 |
| Proteinase 3 | 21.3% (6.6 to 38.1) | 0.005 | 33.2% (17.6 to 50.9) | <0.0001 | 27.2% (10.8 to 46.1) | 0.001 |
| **Systemic inflammation and organ damage** | | | | | |  |
| CD163 | 50.5% (18.5 to 91.1) | 0.001 | 51.0% (21.8 to 87.2) | 0.0002 | 38.6% (10.0 to 74.6) | 0.007 |
| Cardiac Myoglobin | 27.3% (-3.3 to 67.6) | 0.094 | 108.1% (59.8 to 171.0) | <0.0001 | 75.1% (32.6 to 131.2) | 0.0002 |
| TFF3 | 22.9% (8.3 to 39.4) | 0.002 | 49.7% (31.8 to 70.0) | <0.0001 | 35.8% (18.6 to 55.5) | <0.0001 |
| Tenascin C | 33.5% (18.8 to 50.0) | <0.0001 | 45.8% (30.0 to 63.4) | <0.0001 | 34.9% (19.1 to 52.7) | <0.0001 |
| Ferritin | 77.6% (29.3 to 143.9) | 0.0008 | 110.6% (54.5 to 187.2) | <0.0001 | 153.0% (80.7 to 254.3) | <0.0001 |
| TREM1 | 52.2% (33.7 to 73.3) | <0.0001 | 91.3% (67.6 to 118.4) | <0.0001 | 60.2% (40.9 to 82.2) | <0.0001 |
| NTproBNP | 45.5% (-6.4 to 126.2) | **0.102** | 125.6% (48.9 to 241.8) | 0.0002 | 53.1% (3.0 to 127.5) | 0.038 |
| Resistin | 31.4% (9.7 to 57.2) | 0.004 | 75.8% (47.5 to 109.6) | <0.0001 | 52.0% (25.9 to 83.5) | <0.0001 |
| Procalcitonin | 141.9% (61.0 to 263.6) | <0.0001 | 238.6% (130.9 to 396.5) | <0.0001 | 93.8% (33.3 to 181.7) | 0.0009 |
| C-reactive protein | 50.7% (11.8 to 103.0) | 0.009 | 73.8% (31.4 to 129.7) | 0.0002 | 65.6% (22.0 to 124.8) | 0.002 |

Linear regression models were used with log-transformed biomarker concentrations as the outcome and patient group as the main predictor. Analyses were performed with adjustment for plasma creatinine (log-transformed), or for immunosuppression. In a fully adjusted specification, we additionally included demographics (age, sex), vascular comorbidities (prior myocardial infarction, cerebrovascular disease, chronic kidney disease, diabetes), MEWS and care setting in the covariate set. For abbreviations see Table S1.

**Table S6**: Enriched Pathways from Genes with Negative Running Scores in Hemostasis

| **ID** | **Pathway** | **Adjusted p-value** | **Gene Count** | **Gene IDs** |
| --- | --- | --- | --- | --- |
| R-HSA-114604 | GPVI-mediated activation cascade | 0.08 | 21 | *PRKCZ, PIK3R1, PDPN, PIK3CG, PLCG2, SYK, RHOG, PIK3R3, RAC2, PTPN6, RHOA, PIK3R5, VAV2, LCP2, RAC1, PDPK1, VAV1, VAV3, LCK, LYN, FYN* |
| R-HSA-114508 | Effects of PIP2 hydrolysis | 0.08 | 17 | *TRPC3, TRPC7, ITPR3, RASGRP2, DGKA, PRKCE, ITPR1, DAGLA, DGKK, PRKCD, DGKZ, PRKCQ, DGKQ, RASGRP1, DGKD, ABHD6, PRKCH* |

**Figure S1**: Plasma syndecan-1 levels of non-infectious controls and patients with community-acquired pneumonia


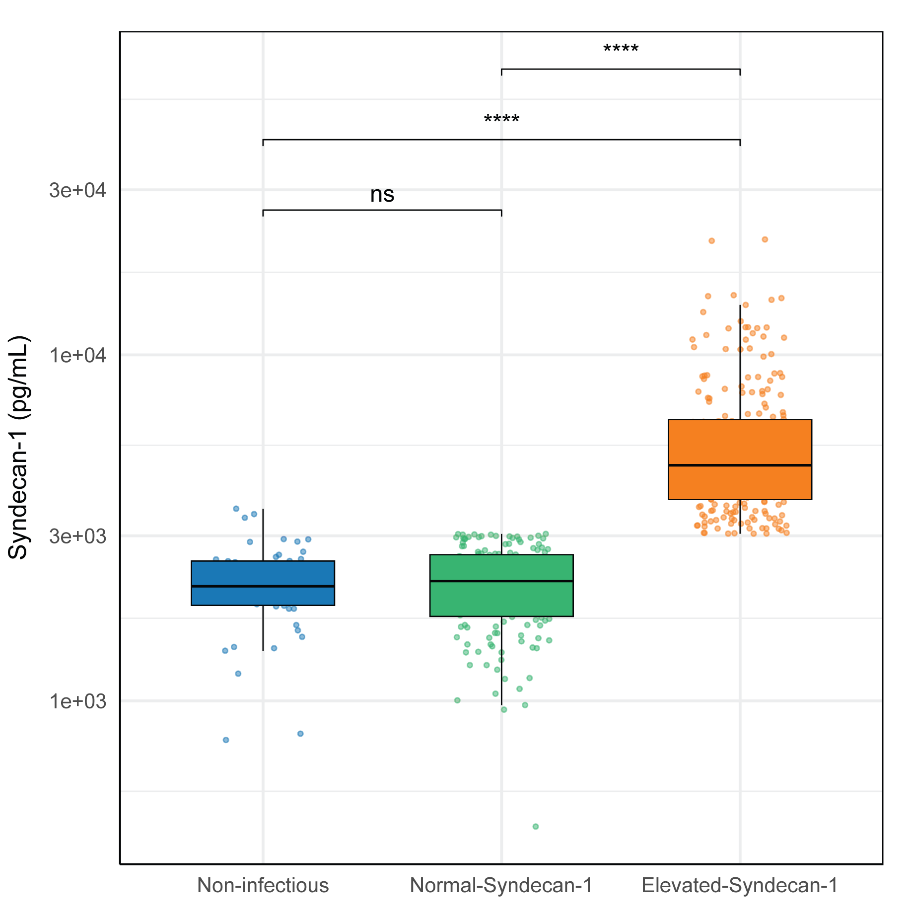


Patients with community-acquired pneumonia were stratified into two groups: the Normal-Syndecan-1 group and the Elevated-Syndecan-1 group based on syndecan-1 levels measured in outpatient controls without infection (see Methods for details). Data are shown as box and whiskers with individual data points. **** p < 0.0001, ns = non-significant.

**Figure S2:** Relationship between syndecan-1 levels and 30-day mortality in unadjusted and adjusted models.


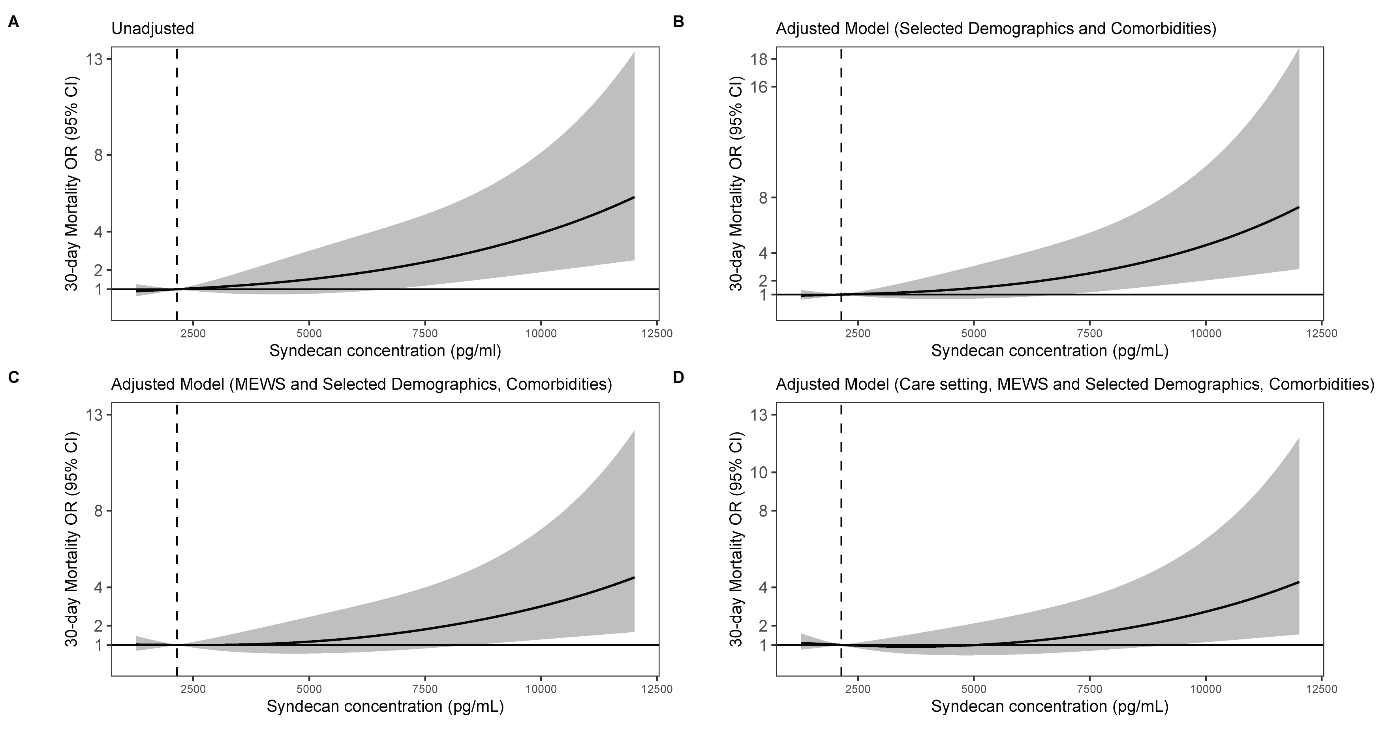


**Legend** **Figure S2:**  **A)** The risk of 30-day mortality was assessed with syndecan-1 treated as a continuous variable. Due to the nonlinear relationship between syndecan-1 levels and mortality, a restricted cubic spline function with three inner knots positioned at default quantile locations was employed. The odds ratio was calculated using the median syndecan-1 level of non-infectious controls as the reference point. **B)** Similar to A), but the 30-day mortality odds ratio was adjusted for demographics (age and sex) as well as vascular comorbidities (including prior myocardial infarction, cerebrovascular disease, chronic kidney disease and diabetes), and immunosuppression.  **C)** Similar to B) with the analysis further adjusted for admission disease severity by incorporating MEWS as an additional confounder. Shaded areas indicate 95% confidence intervals. **D)** Similar to C), with the analysis further adjusted for care setting at sampling to account for baseline risk differences across settings.

**Figure S3:** Kaplan–Meier survival stratified by Syndecan-1 (cut-point via maximally selected rank statistics)


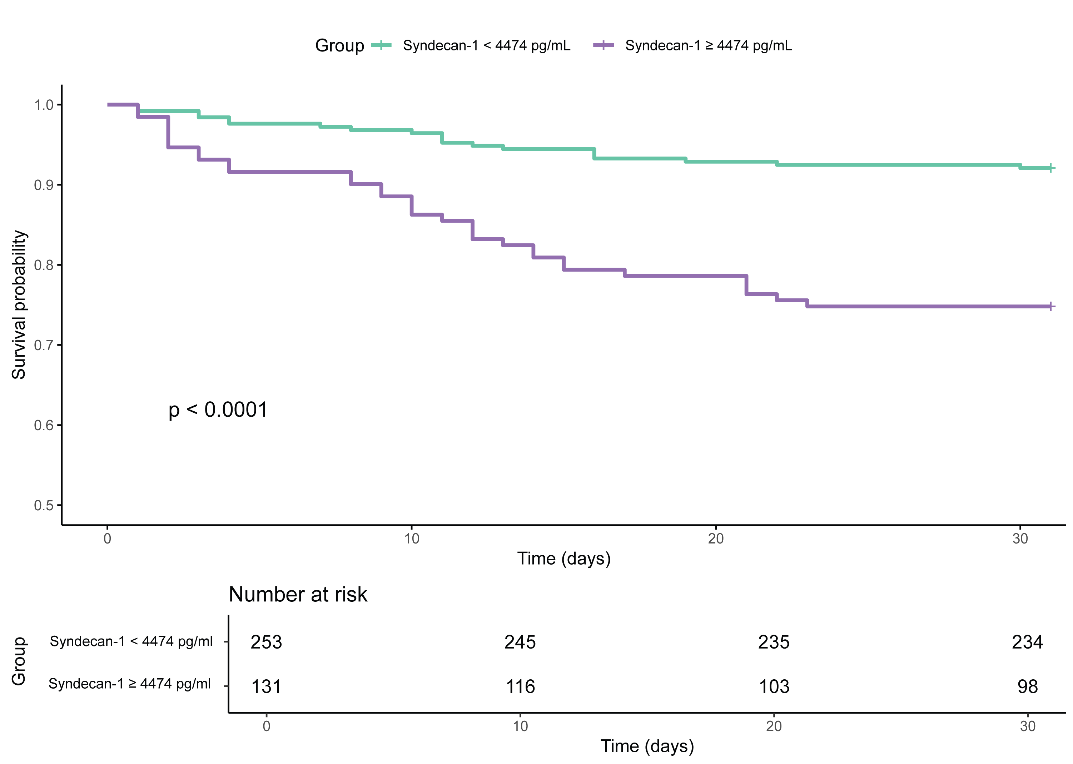


**Figure S4**: Differential expression of host response biomarkers across Syndecan-1 groups defined by a syndecan-1 cutoff of (3351.6 pg/mL; z = 1.96; sensitivity analysis).


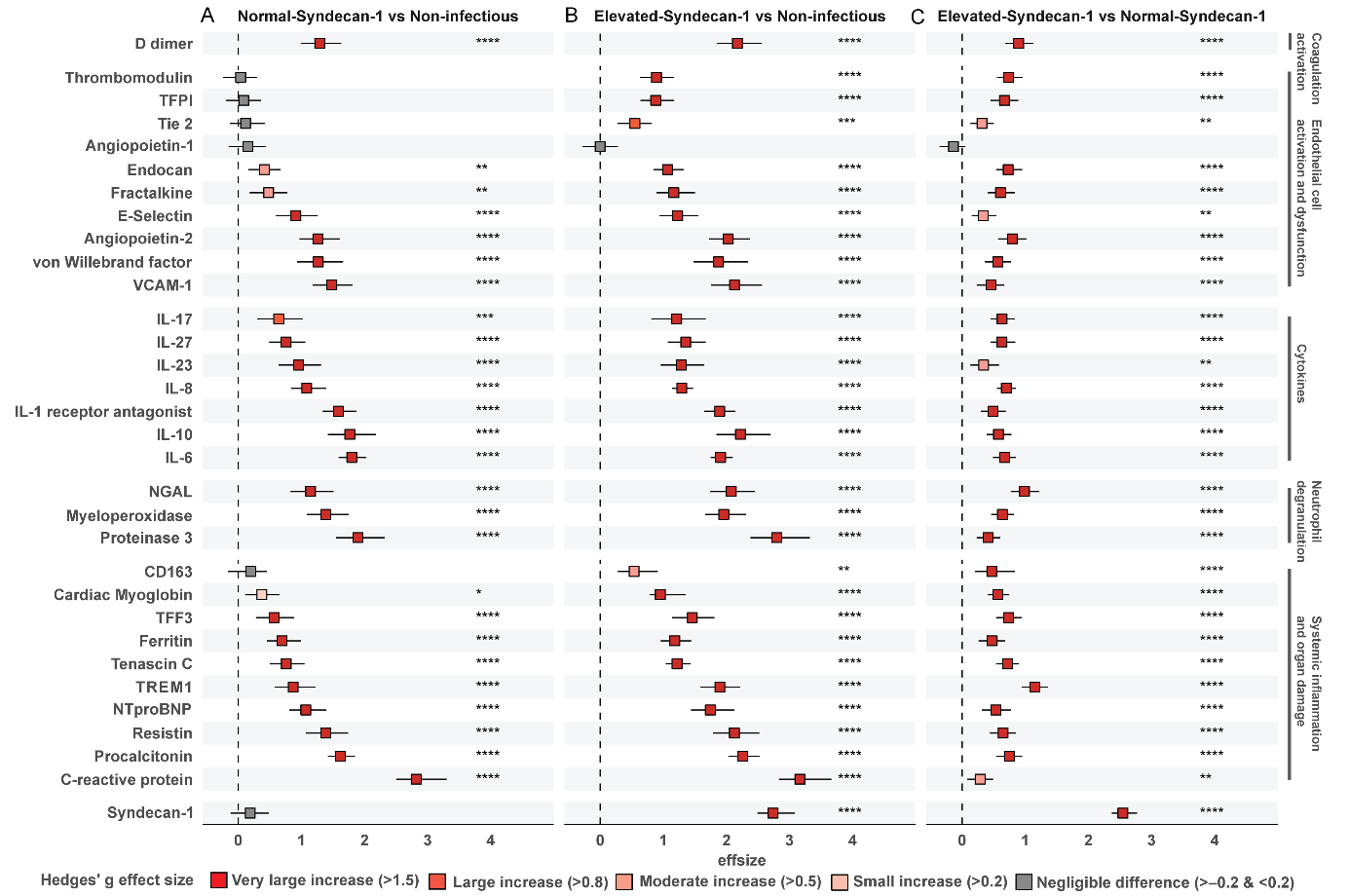


**Legend** **Figure S4:** **A)** Normal-Syndecan-1 vs non-infectious controls, **B)** Elevated-Syndecan-1 vs non-infectious controls, and **C)** Elevated-Syndecan-1 vs Normal-Syndecan-1. Each square represents the Hedges’ g effect size with 95% confidence intervals, where red indicates higher concentrations and blue indicates lower ones. Positive values reflect higher biomarker levels in the first group of the comparison. Biomarkers are organized by biological domain. Color coding indicates both statistical significance and direction of change based on Welch’s t-test with Benjamini-Hochberg correction: red for significant increases, grey for non-significant differences. Hedges g’ effect sizes are considered very large if > 1.5, large if > 0.8, moderate if > 0.5 and small if > 0.2[5]. Abbreviations: IL, interleukin; NGAL, neutrophil gelatinase-associated lipocalin; NTproBNP, aminoterminal pro-B-type natriuretic peptide; TREM1, triggering receptor expressed on myeloid cells 1; VCAM-1, vascular cellular adhesion molecule-1; TFPI, tissue factor pathway inhibitor; TFF3, trefoil factor 3. Note: The syndecan-1 cutoff used in this figure was defined as the non-infectious group mean plus 1.96 standard deviations (sensitivity analysis).

**Figure S5:** Differential gene expression and pathway enrichment analysis comparing the Elevated-Syndecan-1 group with the Normal-Syndecan-1 group defined by a syndecan-1 cutoff of (3351.6 pg/mL; z = 1.96; sensitivity analysis).


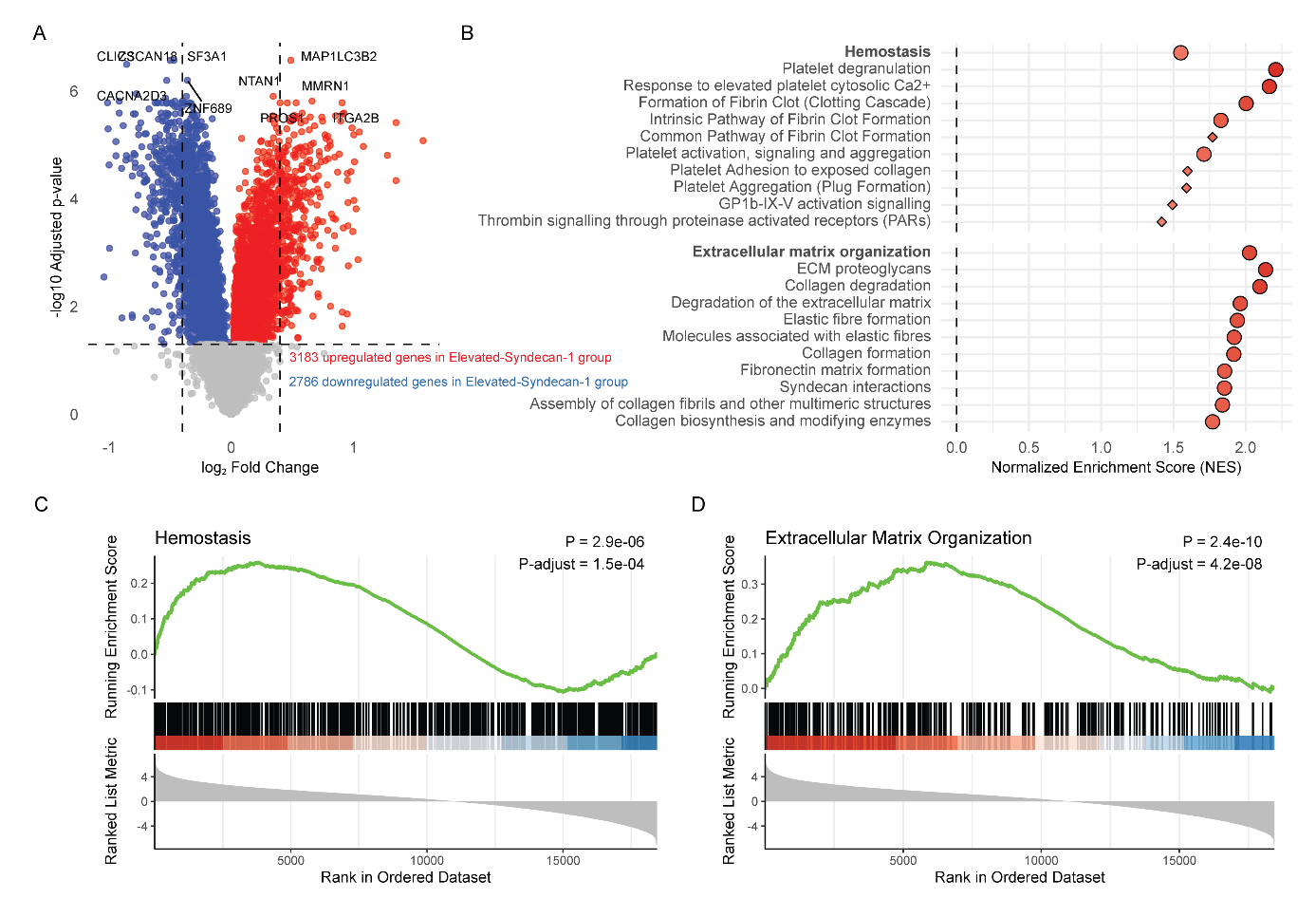


**Legend** **Figure S5:**  **A)** The volcano plot illustrates the differential gene expression between the Elevated-Syndecan-1 group and Normal-Syndecan-1 group . Red points represent 3183 upregulated genes in the Elevated-Syndecan-1 group, while blue points denote 2786 downregulated genes in this group, each meeting an adjusted p-value threshold of less than 0.05. The plot highlights the 10 most significantly upregulated and downregulated genes. **B)** The plot displays the Normalized Enrichment Score (NES) for Reactome pathways enriched in relation to extracellular matrix organization and hemostasis. These parent pathways are further categorized into child pathways (shown in lighter colors). Pathways significantly enriched in the Elevated-Syndecan-1 group are indicated in red. **C)** The green line represents the running enrichment score, reflecting the accumulation of pathway-related genes at the top of the ranked gene list. The peak of this curve marks the point of greatest enrichment. Vertical black lines indicate the rank positions of individual genes within the pathway. The heatmap beneath the plot shows the ranked list metric, with red indicating higher expression and blue indicating lower expression in the Elevated-Syndecan-1 group. **D)** Enrichment plot for the Reactome “Extracellular Matrix Organization” pathway. The syndecan-1 cutoff used was defined as the non-infectious group mean plus 1.96 standard deviations (sensitivity analysis).

**Figure S6:** Expression levels of the 5 most upregulated and the 5 most downregulated genes in the Reactome pathways “Hemostasis” and “Extracellular matrix organization”.


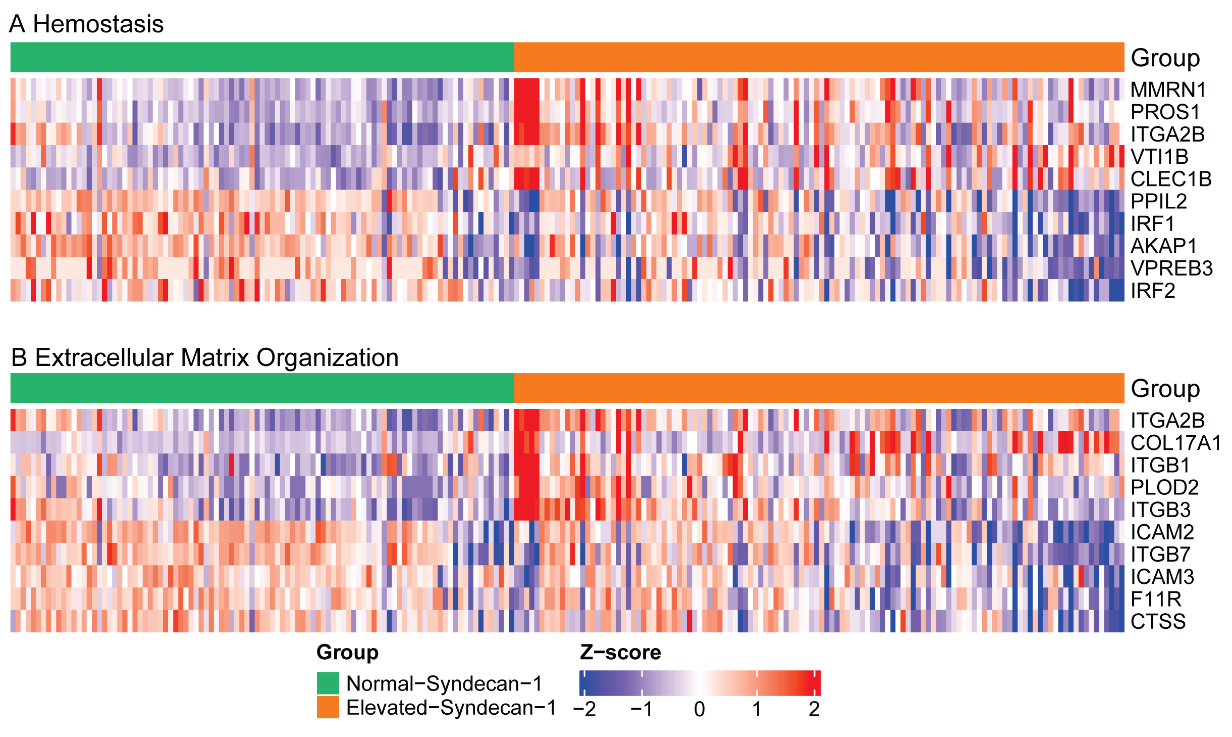


**Legend** **Figure S6:** Each heatmap illustrates the expression levels of the 5 most upregulated genes and the 5 most downregulated genes in one of the three pathways:

**A)** Hemostasis, **B)** Extracellular matrix organization. Red indicates high expression levels, whereas blue indicates low expression levels.
